# Phenotypic characterization of *Neisseria meningitidis* strains isolated from invasive meningococcal disease in Brazil from 2002 to 2017

**DOI:** 10.1099/acmi.0.000079

**Published:** 2019-12-10

**Authors:** Maria Cecília Gorla, Angela Pires Brandao, Juliana Maira Watanabe Pinhata, Camile de Moraes, Gabriela Pereira, Ana Paula Lemos

**Affiliations:** ^1^​ Bacteriology Department, Adolfo Lutz Institute, Av. Dr. Arnaldo 351, São Paulo, CEP 01246-000, SP, Brazil; ^2^​ Oswaldo Cruz Institute, FIOCRUZ, Av. Brasil, 4365, Rio de Janeiro, CEP 21040-900, RJ, Brazil; ^3^​ Secretariat of Health Surveillance, Ministry of Health, Esplanada dos Ministérios, Bloco G, Brasília, CEP 70058-900, DF, Brazil; ^4^​ General Coordination of Laboratories, Ministry of Health, Esplanada dos Ministérios, Bloco G, Brasília, CEP 70058-900, DF, Brazil

**Keywords:** meningococcal surveillance, *Neisseria meningitidis*, laboratory meningococcal surveillance

## Abstract

**Introduction:**

Invasive meningococcal disease (IMD) has a high rate of fatality and may cause severe clinical sequelae. Over the years, the epidemiology of IMD has changed significantly in various regions of the world, and laboratory surveillance of this disease is important for mapping epidemiologic changes.

**Aim:**

To perform phenotypic characterization of *Neisseria meningitidis* strains isolated from invasive disease in Brazil from 2002 to 2017, as a complementation of the data obtained in the period of 1990–2001.

**Methodology:**

In total, 8,689 isolates sent to Adolfo Lutz Institute confirmed as *N. meningitidis* by conventional methods were serogrouped by slide agglutination against MenA, MenB, MenC, MenE, MenW, MenX, MenY and MenZ; serotyped and serosubtyped by a whole-cell dot-blotting assay with monoclonal antibodies.

**Results:**

The isolates were sent from all regions of Brazil, and the southeast region was responsible for the largest number of isolates (57.2 %). Overall, the total sample (*n*=8,689) was represented by serogroups C (*n*=4,729; 54.4 %), B (*n*=3,313; 38.1 %), W (*n*=423; 4.9 %), Y (*n*=203; 2.3 %), X (*n*=5; 0.1 %) and others (*n*=16; 0.2 %). A shift in the prevalence of serogroups was observed in 2006, when serogroup C became the most prevalent (65.5 %), surpassing the serogroup B (21.9 %). The main isolated phenotypes were C:23:P1.14–6; B:4,7:P1.19,15; W:2a:P1.5 and W:2a:P1.5,2.

**Conclusion:**

The data show an important change in the distribution of meningococcal serogroups, serotypes and subtypes occurring during 2002–2017. A continuous laboratory-based surveillance provides robust information to implement appropriate strategies to IMD control.

## Introduction

Invasive meningococcal disease (IMD) is one of the most feared diseases due to its rate of fatality of 5–10 %, and causes severe clinical sequelae such as deafness, amputation and mental impairment [1]. Twelve serogroups of *Neisseria meningitidis* have been characterized based on their capsular polysaccharides, although serogroups A, B, C, W, Y and X are the most clinically significant [2, 3]. Most cases of IMD occur sporadically and vary according to the age, socioeconomic conditions, geographic regions and serogroups involved; outbreaks and epidemics may occur at irregular intervals [4–6]. Since 1975, IMD is a notifiable disease in Brazil and the National Surveillance System (SNV) monitors the epidemiological characteristics of the disease.

From 1990 to 2001, 68, 332 IMD cases were reported to the Brazilian National Notifiable Disease Surveillance System (SINAN), and the annual incidence rate was estimated to be around 1–3 cases per 100, 000 persons [7]; in contrast, in the period from 2002 to 2017, 41, 192 IMD cases were notified and the annual incidence rate was estimated at around 0.55–2.12 cases per 100,000 persons, presenting a clear reduction trend in the period, from 2.12 cases in 2002 to 0.55 cases per 100, 000 in 2017 [8]. Thirty-one percent (*n*=12, 621) of the cases notified between 2002 and 2017 were confirmed by isolation of the causative strain, from which 68.8 % (*n*=8,689) were referred to the National Reference Laboratory for Meningitis at Adolfo Lutz Institute, São Paulo, herein named IAL, for species confirmation and further characterization.

Over the years, the epidemiology of IMD has changed significantly in various regions of the world. The aim of this study was to analyse the phenotypic characteristics of *N. meningitidis* strains causing IMD in Brazil, thus complementing the previously described results [7] of the period from 1990 to 2001.

## Methods

### Meningococcal isolate collection

The IAL receives invasive meningococcal isolates from public health laboratories and hospitals from all Brazilian regions for full phenotypic characterization.

Of the 12, 621 cases of IMD laboratory-confirmed by culture between 2002 and 2017 in Brazil, 8,689 isolates (68.8 %) were sent to IAL.

### Identification and serogrouping of *N. meningitidis*

All the 8,689 IMD isolates were confirmed as *N. meningitidis* by conventional methods (oxidase test and carbohydrate utilization testing for glucose, maltose, sucrose and lactose). Serogrouping was performed by slide agglutination with polyclonal goat or horse antisera, prepared at the IAL against MenA, MenB, MenC, MenE, MenW, MenX, MenY and MenZ, as described previously [9, 10].

### Serotype and serosubtype determination

In order to determine the PorB and PorA types, all the isolates were serotyped and serosubtyped by a whole-cell dot-blotting assay with monoclonal antibodies (mAbs) [11]. The full sets consisted of mAbs for serotypes 1, 2a, 2b, 2 c, 4, 5, 7, 9, 10, 11, 14, 15, 17, 19, 21, 22 and 23, and for serosubtypes P1.1, P1.2, P1.3, P1.4, P1.5, P1.7, P1.9, P1.10, P1.12, P1.14, P1.14–6, P1.15, P1.16, P1.19 and P1.22–1. A set of reference strains of each serotype and serosubtype was used in each reaction as positive and negative controls.

## Results and discussion

Of the 8,689 *N *. *meningitidis* isolates (68.8 %) sent to IAL, 4,969 (57.2 %) were from the Southeast, 1,612 (18.6 %) from the North West, 1,222 (14.1 %) from the South, 596 (6.8 %) from the Central West and 290 (3.3 %) from the North region of Brazil (Table 1). This distribution follows the pattern found in the previous period [7], and is in agreement with the 2010 demographic census, which shows that 42.1 % of the Brazilian population is concentrated in the Southeast region while 8.3 % is in the Northern region (https://censo2010.ibge.gov.br/sinopse/index.php?dados=5&uf=00).

**Table 1.** Distribution of *Neisseria meningitidis* serogroups by geographical regions and by age group, from 2002 to 2017, in Brazil

		2002–2006								2007–2010				
	**B *n* (%)**	**C *n* (%)**	**W *n* (%)**	**Y *n* (%)**	Others* *n* (%)	**Total**	**B *n* (%)**	**C *n* (%)**	**W *n* (%)**	**Y *n* (%)**	Others* *n* (%)	**Total**		
		
**Geographic region**														
North	168 (82.0)	37 (18.0)	–	–	–	205	23 (45.1)	27 (52.9)	1 (2.0)	–	–	51		
North west	607 (75.6)	186 (23.2)	5 (0.6)	5 (0.6)	–	803	128 (25.8)	362 (72.8)	4 (0.8)	3 (0.6)	–	497		
Central west	132 (59.7)	81 (36.7)	4 (1.8)	4 (1.8)	–	221	33 (15.3)	166 (76.9)	12 (5.5)	5 (2.3)	–	216		
South east	871 (45.8)	929 (48.8)	70 (3.7)	30 (1.6)	3 (0.1)	1903	280 (17.8)	1149 (73.0)	101 (6.4)	39 (2.5)	5 (0.3)	1574		
South	387 (74.4)	111 (21.3)	20 (3.9)	2 (0.4)	–	520	152 (56.5)	90 (33.5)	25 (9.3)	2 (0.7)	–	269		
**All regions**	**2165 (59.3)**	**1344 (36.8)**	**99 (2.7)**	**41 (1.1)**	**3 (0.1)**	**3652**	**616 (23.6)**	**1794 (68.8)**	**143 (5.5)**	**49 (1.9)**	**5 (0.2)**	**2607**		
**Age group**														
< 12 mo	309 (59.2)	192 (36.8)	15 (2.9)	6 (1.1)	–	522	100 (30.9)	189 (58.3)	28 (8.6)	7 (2.2)	–	324		
12–23 mo	140 (53.8)	110 (42.3)	8 (3.1)	1 (0.4)	1 (0.4)	260	51 (28.8)	111 (62.7)	9 (5.1)	6 (3.4)	–	177		
24–59 mo	397 (61.2)	228 (35.1)	16 (2.5)	7 (1.1)	1 (0.1)	649	109 (28.4)	260 (67.7)	11 (2.9)	4 (1.0)	–	384		
5–14 y	565 (61.7)	328 (35.8)	17 (1.9)	5 (0.6)	–	915	131 (21.5)	444 (72.8)	25 (4.1)	10 (1.6)	–	610		
15–29 y	347 (61.2)	187 (33.0)	23 (4.0)	10 (1.8)	–	567	87 (18.0)	360 (74.5)	26 (5.4)	9 (1.9)	1 (0.2)	483		
30–49 y	118 (48.6)	114 (46.9)	5 (2.0)	6 (2.5)	–	243	43 (16.1)	206 (77.1)	13 (4.9)	5 (1.9)	–	267		
>= 50 y	64 (55.2)	45 (38.8)	6 (5.1)	1 (0.9)	–	116	32 (20.3)	95 (60.1)	21 (13.3)	7 (4.4)	3 (1.9)	158		
Unknown	225 (59.2)	140 (36.8)	9 (2.4)	5 (1.3)	1 (0.3)	380	63 (30.9)	129 (63.2)	10 (4.9)	1 (0.5)	1 (0.5)	204		
**Total**	**2165 (59.3)**	**1344 (36.8)**	**99 (2.7)**	**41 (1.1)**	**3 (0.1)**	**3652**	**616 (23.6)**	**1794 (68.8)**	**143 (5.5)**	**49 (1.9)**	**5 (0.2)**	**2607**		
				**2011–2017**						**Entire period, 2002–2017**				
	**B *n* (%)**	**C *n* (%)**	**W *n* (%)**	**Y *n* (%)**	**X *n* (%)**	Others* *n* (%)	**Total**	**B *n* (%)**	**C *n* (%)**	**W *n* (%)**	**Y *n* (%)**	**X *n* (%)**	Others* *n* (%)	**Total**

**Geographic region**														
North	10 (29.4)	23 (67.7)	1 (2.9)	–	–	–	34	201 (69.3)	87 (30.0)	2 (0.7)	–	–	–	290
North west	54 (17.3)	223 (71.5)	24 (7.7)	10 (3.2)	–	1 (0.3)	312	789 (48.9)	771 (47.8)	33 (2.1)	18 (1.1)	–	1 (0.1)	1612
Central west	29 (18.2)	112 (70.5)	13 (8.2)	4 (2.5)	–	1 (0.6)	159	194 (32.5)	359 (60.2)	29 (4.9)	13 (2.2)	–	1 (0.2)	596
South east	306 (20.5)	1027 (68.8)	71 (4.8)	81 (5.4)	1 (0.1)	6 (0.4)	1492	1457 (29.3)	3105 (62.5)	242 (4.9)	150 (3.0)	1 (**)	14 (0.3)	4969
South	133 (30.7)	206 (47.6)	72 (16.6)	18 (4.2)	4 (0.9)	–	433	672 (55.0)	407 (33.3)	117 (9.6)	22 (1.8)	4 (0.3)	–	1222
**All regions**	**532 (21.9)**	**1591 (65.5)**	**181 (7.4)**	**113 (4.7)**	**5 (0.2)**	**8 (0.3)**	**2430**	**3313 (38.1)**	**4729 (54.4)**	**423 (4.9)**	**203 (2.3)**	5 (**)	**16 (0.2)**	**8689**
														
**Age group**														
< 12 mo	133 (51.8)	80 (31.1)	35 (13.6)	9 (3.5)	–	–	257	542 (49.1)	461 (41.8)	78 (7.1)	22 (2.0)	–	–	1103
12–23 mo	51 (65.4)	7 (9.0)	15 (19.2)	5 (6.4)	–	–	78	242 (47.0)	228 (44.3)	32 (6.2)	12 (2.3)	–	1 (0.2)	515
24–59 mo	80 (35.4)	110 (48.6)	25 (11.1)	9 (4.0)	2 (0.9)	–	226	586 (46.5)	598 (47.5)	52 (4.1)	20 (1.6)	2 (0.2)	1 (0.1)	1259
5–14 y	98 (17.6)	423 (75.1)	16 (2.8)	23 (4.0)	1 (0.2)	2 (0.3)	563	794 (38.0)	1195 (57.2)	58 (2.8)	38 (1.9)	1 (**)	2 (0.1)	2088
15–29 y	79 (14.6)	414 (76.4)	29 (5.3)	19 (3.5)	–	1 (0.2)	542	513 (32.2)	961 (60.4)	78 (4.9)	38 (2.4)	–	2 (0.1)	1592
30–49 y	46 (11.1)	325 (78.3)	27 (6.5)	15 (3.6)	–	2 (0.5)	415	207 (22.4)	645 (69.7)	45 (4.9)	26 (2.8)	–	2 (0.2)	925
>= 50 y	33 (11.3)	192 (66.0)	31 (10.7)	30 (10.3)	2 (0.7)	3 (1.0)	291	129 (22.8)	332 (58.8)	58 (10.3)	38 (6.7)	2 (0.3)	6 (1.1)	565
Unknown	12 (20.7)	40 (68.9)	3 (5.2)	3 (5.2)	–	–	58	300 (46.7)	309 (48.2)	22 (3.4)	9 (1.4)	–	2 (0.3)	642
**Total**	**532 (21.9)**	**1591 (65.5)**	**181 (7.4)**	**113 (4.7)**	**5 (0.2)**	**8 (0.3)**	**2430**	**3313 (38.1)**	**4729 (54.4)**	**423 (4.9)**	**203 (2.3)**	5 (**)	**16 (0.2)**	**8689**

*Serogroup E, not serogrouped or polyagglutinable.

**Number of isolates too small to calculate a percentage.

The isolates were recovered from normally sterile sites, i.e. cerebrospinal fluid (*n*=6,493; 74.7 %), blood (*n*=2,186; 25.2 %), and others (*n*=10; 0.1 %) (data not shown).

**Fig. 1.**
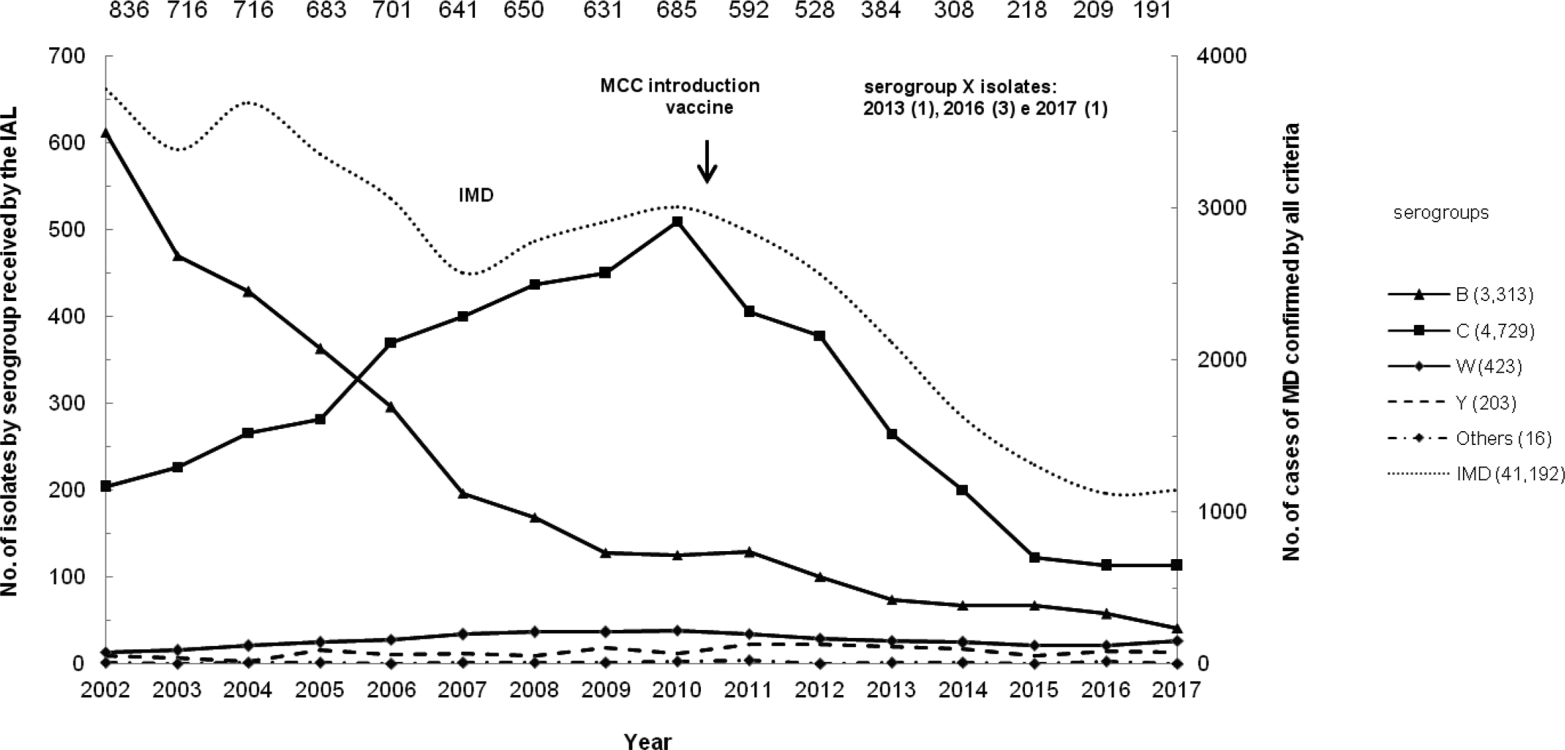
Number of *Neisseria meningitidis* isolates from invasive meningococcal disease in Brazil by serogroup per year, 2002–2017. The total number of isolates for each year is shown above the respective data points.

The distribution of isolates by serogroup is shown in Fig. 1, and is similar to the distribution curve of IMD cases reported over this period. Overall, the total sample (*n*=8,689) was represented by serogroups C (*n*=4,729; 54.4 %), B (*n*=3,313; 38.1 %), W (*n*=423; 4.9 %), Y (*n*=203; 2.3 %), X (*n*=5; 0.1 %) and others (*n*=16; 0.2 %).

Serogroup B has been the main cause of IMD in Brazil since 1980, with B:4,7:P1.19,15-ST-32 clonal complex (CC) as the most frequent phenotype isolated at all ages until 2002. Serogroup B remains present in all regions of Brazil, with the clone B:4,7:P1.19,15 still being the most prevalent (56 %) among the serogroup B strains typed [7, 12]. However, from 2002 the incidence of serogroup B began to decline, and, in 2006, it was surpassed by a new emerging meningococcal serogroup C clone, C:23:P1.14–6. This serogroup C belonging to ST-103 CC, was described for the first time associated with an epidemic situation in Brazil [13]. Isolates of MenC-ST-103 CC, belonging exclusively to ST-5133, were the most common amongst MenC in Poland in 2005 and constituted 42 % of all MenC in the period of 2009–2011, but the outbreaks notified there, between 2002 and 2011, were all caused by MenC of ST-11 CC [14]. The ST-103 CC has been also described among sporadic cases of IMD by serogroup C in the Czech Republic accounting for less than 1 % of serogroup C isolates [15]. In Brazil, this new clone MenC-ST-103, represented by the main STs: ST-3779, ST-3780, ST-5122 and ST-5123, has been responsible for several IMD outbreaks in the period 2003 to 2010, reaching its highest incidence rate in 2010 as 0.62 per 100,000, significantly higher than the 0.10 per 100,000 for serogroup B in this same year [16–20]. The shift from serogroup B to serogroup C was first detected in the Southeast region (2002–2006) followed by the North West and Central West regions (2007–2010), and finally by the North and South regions (2011–2017) (Table 1). This new MenC clone remains currently the main cause of IMD in Brazil, although with a lower incidence rate of 0.17 per 100,000 in 2017, due to the introduction in 2010 of a Meningococcal C Conjugate Vaccine (MCC vaccine) into the national immunization program. Initially, the strategy was to vaccinate children at 3 and 5 months, with a booster dose at 12 months of age, and no catch-up campaigns. After implementation of this vaccine, the IMD overall rate has decreased from 1.54 per 100,000 in 2010 to 0.55 per 100,000 in 2017 [8]. As shown in Table 1, there has been a substantial reduction in the frequency of serogroup C isolates from infants and older children, but the prevalence of serogroup C isolates remained relatively high in adolescents and young adults. As suggested by Andrade *et al*. [21], the reduction of IMD cases in children was mostly due to the direct effect of the vaccination. To provide protection for the older groups, serogroup C vaccination for adolescents aged 12 to 13 years was implemented in March 2017, and by 2020 the age range will be gradually increased, starting at 9 years of age [21]; http://www.aids.gov.br/pt-br/legislacao/nota-informativa-no-3112016cgpnidevitsvsms.

Although serogroup C remains prevalent in all regions of Brazil, the increased incidence of serogroup W in the Southern states from 2014 is of concern because of its potential to cause epidemics, severe disease and its high case fatality rate as has been seen in other countries [22, 23] (Table 1). The clone W:2a:P1.5,2-ST-11 CC, called ‘South American strain’, emerged in 2003 in Southern Brazil [24]. Although it is characterized as ST11/ET37 with similar phenotypic characteristics as to the isolates associated with Hajj 2000, a whole genome sequencing (WGS) analysis demonstrated that the MenW:CC11 ‘South American strain’ is distinct from the Hajj outbreak strain [25]. The ‘South American strain’ has spread to Argentina, Chile, England and Wales, where they became endemic after undergoing diversification in a sublineage named the 2013-strain [26].

Serogroup Y has been most commonly related to carriage isolates over time [27, 28] but this situation has changed in many countries like the USA, for example, where it has become (during the latter half of the 1990s) an important cause of IMD. The increase in the prevalence of serogroup Y in the USA is attributed to the emergence of a ST-23 clone and, while the incidence of all serogroups has fallen in the USA in recent years, serogroup Y continues to be responsible for approximately a third of endemic disease cases [29]. The proportion of MenY disease has also increased during the last years in some European countries such as Sweden, Finland, England and Wales, which may be in part due to the use of MenC conjugate vaccine as opposed to the quadrivalent serogroup ACWY vaccine [3]. The most frequent genotype that has been described is Y:P1.5–2,10-1 CC-23 [30, 31].

Many countries in Central and South America have experienced an increasing incidence of IMD caused by serogroup Y, such as Costa Rica, Colombia, Venezuela, Argentina, Chile and Uruguay. These strains belong mainly to ST-23 CC, similar to those prevalent in the USA [32–34].

In Brazil, the incidence of serogroup Y presented a slight increase in the Southeast region, in the period 2011–2017, but in cumulative terms, from 2002 to 2017, it was recovered from about 2.3 % of all IMD isolates (Table 1). A study carried out by Abad *et al*. [33] with 45 serogroup Y strains from Brazil, isolated in the period of 2000–2006, showed that two main clones, ST-23 and ST-5770, accounted for 35.6 and 48.9 % of the isolates, respectively.

In contrast to the sub-Saharan Africa, where MenX has caused outbreaks in several countries since 2006 [35–37], developed countries have reported only sporadic cases of serogroup X IMD. In Brazil, serogroup X was first recovered from an IMD case in 2013 in the Southeast region, at the São Paulo state. Since then, another three cases occurred in the Southern states Rio Grande do Sul and Santa Catarina in 2016, and one more in the São Paulo state in 2017, isolated from bronchial washing fluid; all of them belonging to the clone X:4,7:P1.19,15-ST-2888 (unpublished data, personal communication, Lemos AP), the same clone that has been described in Italy [38]. Interestingly, the MenX clone X:NT:P1.5–1-CC-181, the most prevalent in recent IMD epidemic outbreaks in many African regions [37], was not recovered in Brazil despite the significant movement of migrants from Africa to southern Brazil (Rio Grande do Sul state) since 2013 (https://gauchazh.clicrbs.com.br/geral/noticia/2014/08/Novos-imigrantes-mudam-o-cenario-do-Rio-Grande-do-Sul-4576728.html).

**Fig. 2.**
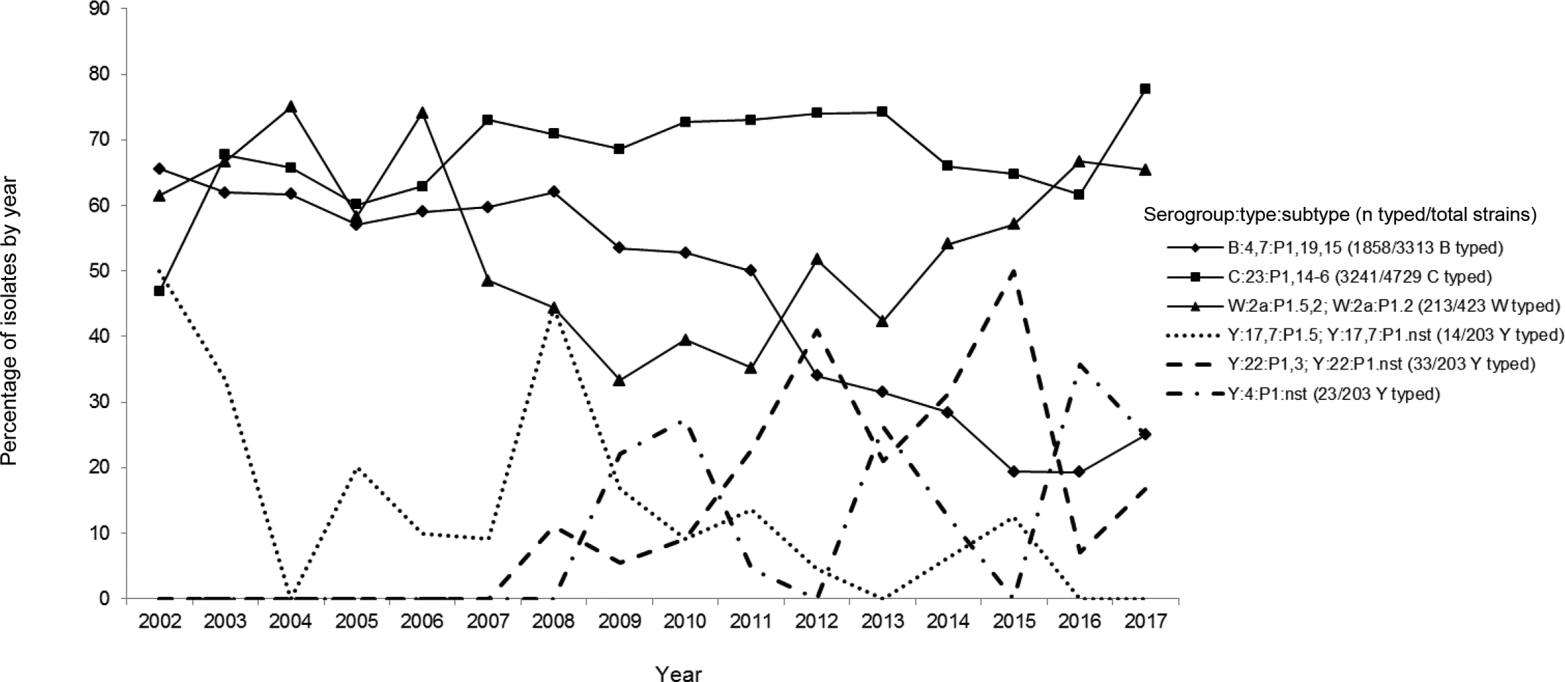
Annual proportion of the main phenotypes of *N. meningitidis* from invasive meningococcal disease in Brazil, 2002–2017. The percentage refers to the number of strains in a given serogroup that were further typed and subtyped.

Various phenotypes were recovered over the period of 2002–2017, and the prevalent ones are shown in Fig. 2. The serotype and serosubtype distributions of meningococcal isolates are shown in Tables 2 and 3, respectively. Serogroup B isolates displayed the highest diversity of phenotypes (202 different serotype:serosubtype compositions, data not shown), but B:4,7:P1.19,15 remains the most prevalent among serogroup B isolates (1,858/3,313; 56 %) ever since 1980. Serotype 19 was the second most prevalent serotype linked to serogroup B strains, increasing slightly from 2007, contributing in the whole period with 18 % of the B serotypes. In a previous study on antimicrobial resistance of meningococcal strains from the period of 2009–2016, we identified that the vast majority of serotype 19 isolates linked to serogroup B showed intermediate resistance to penicillin [39].

**Table 2.** Serotype distribution of *N meningitidis* isolates by the main serogroups (B, C, W, Y, X), Brazil 2002–2017

	2002–2006				2007–2010				2011–2017				
**Serotype**	**B**	**C**	**w**	**Y**	**B**	**C**	**w**	**Y**	**B**	**C**	**w**	**Y**	**X**
	***n* (%)**	***n* (%)**	***n* (%)**	***n* (%)**	***n* (%)**	***n* (%)**	***n* (%)**	***n* (%)**	***n* (%)**	***n* (%)**	***n* (%)**	***n* (%)**	***n* (%)**
2a	16 (0.7)	68 (5.0)	78 (78.9)	2 (4.9)	1 (0.2)	135 (7.5)	82 (57.3)	1 (2.0)	3 (0.6)	87 (5.5)	112 (61.9)	4 (3.5)	–
2b	2 (0.1)	43 (3.2)	11 (11.1)	–	–	19 (1.1)	52 (36.4)	–	–	9 (0.6)	52 (28.7)	3 (2.7)	–
2c	–	–	–	–	–	2 (0.1)	1 (0.7)	–	–	–	–	2 (1.8)	–
4	30 (1.4)	14 (1.0)	–	–	12 (2.0)	–	–	7 (14.3)	18 (3.4)	–	–	18 (16.0)	–
4,7	1698 (78.5)	87 (6.5)	3 (3.0)	3 (7.3)	433 (70.3)	30 (1.7)	1 (0.7)	2 (4.1)	255 (47.9)	18 (1.1)	–	4 (3.5)	5 (100)
4,10	54 (2.5)	4 (0.3)	1 (1.0)	–	3 (0.5)	–	1 (0.7)	–	10 (1.9)	–	–	4 (3.5)	–
4,14	–	–	–	1 (2.4)	6 (0.9)	–	–	4 (8.2)	–	–	–	–	–
14	16 (0.7)	1 (0.1)	–	2 (4.9)	–	1 (0.05)	–	1 (2.0)	–	–	–	–	–
15	86 (4.0)	–	–	1 (2.4)	18 (2.9)	–	–	1 (2.0)	6 (1.1)	–	–	1 (0.9)	–
17,7	44 (2.0)	4 (0.3)	–	14 (34.2)	9 (1.5)	1 (0.05)	–	9 (18.4)	9 (1.7)	5 (0.3)	–	6 (5.3)	–
19	30 (1.4)	6 (0.5)	1 (1.0)	–	21 (3.4)	–	–	3 (6.1)	96 (18.0)	3 (0.2)	–	8 (7.1)	–
19,1	41 (1.9)	1 (0.1)	–	2 (4.9)	36 (5.8)	2 (0.1)	–	–	44 (8.3)	1 (0.05)	–	8 (7.1)	–
19,7	29 (1.3)	3 (0.2)	–	3 (7.3)	14 (2.3)	1 (0.05)	–	–	27 (5.1)	3 (0.2)	–	1 (0.9)	–
19,10	26 (1.2)	1 (0.1)	1 (1.0)	2 (4.9)	4 (0.7)	1 (0.05)	2 (1.4)	2 (4.1)	2 (0.4)	–	1 (0.5)	7 (6.2)	–
19,14	23 (1.1)	–	–	11 (26.8)	13 (2.1)	3 (0.2)	–	5 (10.2)	–	–	–	–	–
22	–	1 (0.1)	–	–	–	2 (0.1)	1 (0.7)	3 (6.1)	1 (0.2)	1 (0.05)	–	31 (27.4)	–
23	7 (0.3)	1057 (78.6)	1 (1.0)	–	7 (1.1)	1524 (84.9)	1 (0.7)	–	14 (2.6)	1397 (87.8)	5 (2.8)	–	–
nt	63 (2.9)	54 (4.0)	3 (3.0)	–	39 (6.3)	73 (4.1)	2 (1.4)	11 (22.5)	47 (8.8)	67 (4.2)	11 (6.1)	16 (14.1)	–
**Total**	**2165**	**1344**	**99**	**41**	**616**	**1794**	**143**	**49**	**532**	**1591**	**181**	**113**	**5**

**Table 3.** Serosubtype distribution of *N meningitidis* isolates by the main serogroups (B, C, W, Y, X), Brazil 2002–2017

2002–2006					2007–2010				2011–2017				
**Serosubtype**	**B**	**C**	**w**	**Y**	**B**	**C**	**w**	**Y**	**B**	**C**	**w**	**Y**	**X**
	***n* (%)**	***n* (%)**	***n* (%)**	***n* (%)**	***n* (%)**	***n* (%)**	***n* (%)**	***n* (%)**	***n* (%)**	***n* (%**)	***n* (%**)	***n* (%**)	***n* (%**)
P1.1	1 (0.05)	–	–	–	2 (0.3)	5 (0.3)	–	–	4 (0.8)	–	–	–	–
P1.2	12 (0.6)	10 (0.7)	61 (61.6)	2 (4.9)	2 (0.3)	13 (0.7)	59 (41.2)	6 (12.3)	1 (0.2)	2 (0.1)	41 (22.6)	–	–
P1.3	52 (2.4)	15 (1.2)	–	–	17 (2.8)	5 (0.3)	3 (2.1)	4 (8.2)	5 (0.9)	2 (0.1)	7 (3.9)	19 (16.8)	–
P1.4	6 (0.3)	3 (0.2)	–	–	5 (0.8)	2 (0.1)	–	–	16 (3.0)	10 (0.6)	–	1 (0.9)	–
P1.5	25 (1.2)	55 (4.1)	2 (2.0)	18 (43.9)	7 (1.1)	126 (7.0)	9 (6.3)	7 (14.3)	7 (1.3)	81 (5.1)	4 (2.2)	11 (9.7)	–
P1.5,2	4 (0.2)	49 (3.7)	17 (17.2)	3 (7.3)	2 (0.3)	17 (1.0)	37 (25.9)	5 (10.2)	4 (0.8)	12 (0.8)	104 (57.5)	13 (11.5)	–
P1.7	19 (0.9)	3 (0.2)	–	–	9 (1.5)	1 (0.05)	–	1 (2.0)	7 (1.3)	6 (0.4)	–	–	–
P1.7,1	224 (10.3)	30 (2.2)	–	1 (2.4)	28 (4.5)	11 (0.6)	–	–	7 (1.3)	3 (0.2)	–	–	–
P1.7,16	68 (3.1)	–	–	–	9 (1.5)	–	–	–	5 (0.9)	–	–	–	–
P1.9	57 (2.6)	10 (0.7)	–	–	12 (2.0)	1 (0.05)	–	–	12 (2.3)	3 (0.2)	–	10 (8.8)	–
P1.10	1 (0.05)	12 (0.9)	–	2 (4.9)	–	2 (0.1)	–	–	–	–	–	2 (1.8)	–
P1.14	24 (1.1)	–	–	–	5 (0.8)	–	–	–	35 (6.6)	–	–	–	–
P1.14–6	10 (0.5)	870 (64.7)	–	–	11 (1.8)	1,365 (76.1)	–	–	21 (3.9)	1,198 (75.3)	–	–	–
P1.16	15 (0.7)	–	1 (1.0)	–	6 (1.0)	3 (0.2)	7 (4.9)	1 (2.0)	9 (1.7)	–	–	2 (1.8)	–
P1.19,15	1,468 (67.8)	63 (4.7)	5 (5.1)	–	397 (64.5)	18 (1.0)	–	1 (2.0)	224 (42.1)	17 (1.1)	–	1 (0.9)	5 (100)
P1.22–1,14	35 (1.6)	4 (0.3)	–	–	15 (2.4)	–	–	–	9 (1.7)	–	–	–	–
nt	144 (6.6)	220 (16.4)	13 (13.1)	15 (36.6)	89 (14.4)	225 (12.5)	28 (19.6)	24 (49.0)	166 (31.2)	257 (16.1)	25 (13.8)	54 (47.8)	–
**Total**	**2,165**	**1,344**	**99**	**41**	**616**	**1,794**	**143**	**49**	**532**	**1,591**	**181**	**113**	**5**

Serogroup C isolates displayed 98 phenotypes, and the most prevalent were 23:P1.14–6 (3,241/4,729; 68.5 %) followed by 23:P1.nst (569/4,729; 12 %) and 2a:P1.5 plus 2a:P1.5,2 (201/4,729; 4.2 %). The clone C:23:P1.14–6 emerged in 2002, and remains prevalent today.

Serogroup W showed 34 phenotypes, of which 2a:P1.5 plus 2a:P1.5,2 was the most prevalent (213/423; 50.2 %), followed by 2b:P1.5 plus 2b:P1.5,2 (92/423; 21.7 %). Although the isolates were not typed molecularly, the prevalence of W:2a, a surrogate marker, suggests the prevalence of the hyper virulent W:2a ST-11 CC causing IMD in this period in Brazil [40]. As reported by other authors, the fatality rate for group W IMD has been high also in Brazil, ranging from 17 to 37 % between 2010 and 2017 [8, 22, 23, 40, 41].

Serogroup Y displayed 55 phenotypes, and the following three were the most prevalent: 22:P1.3 plus 22:P1.nst (33/203; 16.3 %), 4:P1.nst (23/203; 11.3 %) and 17,7:P1.5 plus 17,7:P1.nst (14/203; 6.9 %).

As presented in Tables 2 and 3, serogroup X showed a single phenotype, 4,7:P1.19,15 (5/5; 100 %).

### Conclusion

The data presented herein show an important change in the IMD epidemiological scenario due to a significant shift from serogroup B to serogroup C observed from 2006 onwards, when serogroup C became the most frequent capsular type.

Performing a continuous laboratory-based surveillance highlighting the changes over time in serogroups and serotypes:serosubtypes distribution of invasive *N. meningitidis* contributes to the knowledge of the IMD epidemiology in Brazil, providing robust information to implement appropriate strategies to the IMD control.
